# Resolution of cell-mediated airways diseases

**DOI:** 10.1186/1465-9921-11-75

**Published:** 2010-06-11

**Authors:** Carl G Persson, Lena Uller

**Affiliations:** 1 Department of Clinical Pharmacology, Lund University Hospital, S-22185 Lund, Sweden; 2 Department of Experimental Medical Science BMC D12, Lund University, Lund, S-22184 Sweden

## Abstract

"Inflammation resolution" has of late become a topical research area. Activation of resolution phase mechanisms, involving select post-transcriptional regulons, transcription factors, 'autacoids', and cell phenotypes, is now considered to resolve inflammatory diseases. Critical to this discourse on resolution is the elimination of inflammatory cells through apoptosis and phagocytosis. For major inflammatory diseases such as asthma and COPD we propose an alternative path to apoptosis for cell elimination. We argue that transepithelial migration of airway wall leukocytes, followed by mucociliary clearance, efficiently and non-injuriously eliminates pro-inflammatory cells from diseased airway tissues. First, it seems clear that numerous infiltrated granulocytes and lymphocytes can be speedily transmitted into the airway lumen without harming the epithelial barrier. Then there are a wide range of 'unexpected' findings demonstrating that clinical improvement of asthma and COPD is not only associated with decreasing numbers of airway wall inflammatory cells but also with increasing numbers of these cells in the airway lumen. Finally, effects of inhibition of transepithelial migration support the present hypothesis. Airway inflammatory processes have thus been much aggravated when transepithelial exit of leukocytes has been inhibited. In conclusion, the present hypothesis highlights risks involved in drug-induced inhibition of transepithelial migration of airway wall leukocytes. It helps interpretation of common airway lumen data, and suggests approaches to treat cell-mediated airway inflammation.

## Introduction

Mechanisms active in development of cell-mediated airways disease such as asthma and chronic obstructive pulmonary disease (COPD) may differ from mechanisms involved in exacerbations of these diseases. Different mechanisms again would be involved in resolution of inflammation and healing of the diseased airways. A major aspect of resolution is the elimination of inflammatory cells from the diseased airway wall. This is accomplished, it is thought, by activation of a programmed cell death (apoptosis) followed by 'silent' elimination through phagocytosis of the apoptotic cells. Based on their potential to induce apoptosis of eosinophils and lymphocytes, and increase phagocytosis of apoptotic leukocytes, the mainstay airway anti-inflammatory drugs, glucocorticoids, are considered as pro-resolution drugs ([1], and references cited therein). However, it appears that few in vivo data have been publicised during the last two decades in support of a significant role of leukocyte apoptosis in airways diseases, whether steroid treatment has been involved or not. This limited support for a central dogma on resolution may increasingly be realised by authors involved in research on respiratory disorders: Downey et al [2] recently observed that findings of reduced neutrophil apoptosis in resolving exacerbations of cystic fibrosis "seem counter intuitive as it should be expected that neutrophil apoptosis should have increased to aid resolution of infection and inflammation". On a slightly different note Porter [3], examining transepithelial migration of lymphocytes in vitro, stated that it is widely assumed that the clearance of these cells from inflamed airway tissues involves apoptosis thus "ignoring a potentially very important exit across the bronchial epithelial barrier". This exit has been named 'luminal entry'. Analogous to the exit of cells across the venular endothelial barrier it may also be called 'transepithelial egression', 'transepithelial migration', or 'transmigration'.

Here we discuss the possibility that transepithelial migration of infiltrated airway wall leukocytes is important for resolution of airway inflammation. The present review is guided primarily by actual, independent in vivo observations [4-6]. As such it may differ dramatically from current mechanism-driven approaches by which in vivo observations, too uncritically, may have to comply with the accepted dogma. After introductory paragraphs on development of the present hypothesis and on the rapidly growing interest in resolution of inflammation, we discuss flaws in the studies that have suggested that apoptosis/phagocytosis are key drivers for inflammation resolution in airways diseases. Then we provide a large amount of circumstantial evidence in support of the alternative concept of transepithelial migration/mucociliary clearance as a means of inflammation resolution. Our focus is on observations in patients with inflamed airways. This approach is complemented by in vivo data generated in animal models on inflammation resolution and its inhibition. Reflecting the current lack of an accepted research paradigm in the field, mechanisms involved in transepithelial migration have rarely been explored as a mode of resolving airway tissue inflammation. This state of the art is reflected in the present review by a frugal account of in vitro observations. It is largely for future studies to delineate details of molecular regulation of elimination of leukocytes by their migration through airway tissue components and across the epithelial lining.

## Development of a hypothesis

Together with Jonas Erjefält we have examined numerous airway tissues in health and disease without being able to support the proposed role of granulocyte apoptosis. Instead our work led to the identification of primary cytolysis, without prior apoptosis, as an in vivo paradigm for eosinophil death in the human airway wall [7,8]. This fate had little to do with resolution but was a mode of cell activation causing the release of clusters of free eosinophil granules [7,8]. For non-injurious elimination of airway wall eosinophils we had to look elsewhere. The old literature on asthma [9] was somewhat helpful. Around the turn of the 19^th ^century it was noted that profuse sputum eosinophilia accompanied the clinical improvement of severe asthma. Hence, there is nothing novel in the thinking that elimination of numerous leukocytes from diseased airways can occur via the airway lumen route.

Initially we hypothesised that transepithelial migration was one of several modes of elimination of airway wall eosinophils along with apoptosis and cytolysis [7,10]. Further unexpected failures to detect apoptotic cells in vivo made it apparent that the transepithelial pathway could be the major mode of elimination of airway wall granulocytes [6,8,11-13]. Similarly, the unexpected failure to detect oedema at extravasation of plasma in the airway mucosa had once led to the observation that extravasated, non-sieved plasma could swiftly disappear from the airway wall by moving across an intact epithelial lining [14]. Aiding the transepithelial route of cell and protein elimination, the airway epithelium strongly favours the passage of leukocytes and plasma in the physiological basal to apical direction [14-20].

Both infiltrated leukocytes and extravasated plasma proteins can be transported by lymph flow but this is an exceedingly slow elimination process compared to the exit through transepithelial egression-exudation [13,21]. As demonstrated by Lehman et al [20] for lymphocytes, cells can move from the airway lumen across the alveolar (not the bronchial) epithelium, migrate to regional lymph nodes, and rejoin the systemic systems. In allergen-challenged mice lung regional lymph nodes become heavily infiltrated also with eosinophils [22] but this pathway can explain only a minor part of the disappearance of these cells from the airways [13]. Experiments by Buckley et al [23] and McGettrick et al [24] involving endothelial cell monolayers suggest the additional possibility that transmigrated neutrophils and lymphocytes, perhaps lymphocytes more than neutrophils, to varying degrees can transmigrate back in the reverse direction. If translated to in vivo these data mean that leukocytes could leave the inflamed airway tissue by reverse endothelial transmigration. The importance of this possibility is not known. In their recent editorial on resolution of inflammation, Haworth and Buckley [25] do not mention reverse endothelial migration of leukocytes.

Our continued studies of airways in vivo have specifically involved the early resolution phase as well as steroid treatment in animals and man [26,27] but the findings do not support the assumed role of apoptosis [28-30]. For example, five days' topical steroid treatment of individuals with allergic rhinitis reduced the subepithelial eosinophilia more than the epithelial eosinophilia. This effect agreed with the possibility of eosinophils trafficking towards the airway lumen. Further, at this stage of a rapidly resolving eosinophilic allergic inflammation no apoptotic eosinophil (phagocytosed or non-phagocytosed) was detected in the airway tissue. The allergic inflammation had evoked a general increase of apoptotic cells in the diseased airway mucosa and these non-eosinophilic apoptotic cells were significantly reduced by the steroid treatment, as were several other tissue signs of allergic inflammation [27]. As discussed below a wide range of clinical reports have failed to demonstrate clear roles of leukocyte apoptosis in airways diseases. We further note that many clinical observations, previously discussed as unexpected and puzzling data, actually support the transepithelial migration mode of resolving cell-mediated airways disease. This particular role of transepithelial egression also helps explain significant findings in animal model studies. The present hypothesis needs consideration in current development of novel drugs that affect leukocyte trafficking. These aspects make it timely to review the area.

## Multifaceted interest in resolution of inflammation

Infections, allergic reactions and a variety of other insults cause inflammation. Inflammation resolution involves normalisation of microcirculatory activities, loss of infiltrated cells, and healing of any injury that may have occurred. The resolution may not mean that everything returns to homeostasis because long after the inflammation has resolved significant changes in the innate responsiveness of cells such as the asthmatic epithelium may linger to meet the next insult somewhat differently [31,32]. It is well known that individual mediators, cytokines, and cells may have both pro- and anti-inflammatory facets. Recently, there have been intriguing attempts at identifying endogenous agents with particularly active roles in resolution of inflammation. Thus, there is focus on pro-resolution effects of IL-10 and TGF-beta, adenosine and prostaglandin D2, lipoxins and other lipid mediators [1,33-35]. Some of these regulatory molecules may live up to the actual meaning of the now almost obsolete name 'autacoids' (self remedy). Important roles of certain cells and cell phenotypes in resolution are also entertained with focus on regulatory T cells, macrophages, and neutrophils [1,35]. In addition, select transcription factors [36] and post-transcriptional regulons [37] are given roles in resolution of inflammation. This development has brought resolution hypotheses to the forefront of discussions of airways disease mechanisms [34,38]. Common to the rapidly growing, multifaceted literature on mechanisms of resolution of inflammatory disease processes is the centrality of leukocyte apoptosis followed by phagocytosis of the apoptotic leukocytes. Apoptosis mechanisms are consistently emphasized whereas it appears that a role of transepithelial cell migration may have been overlooked.

### Role of leukocyte apoptosis in airway lumen?

Apoptosis of leukocytes in the more accessible airway lumen has been studied with the assumption that the findings are relevant for cells in the airway wall. However, clear distinction between findings in the airway lumen and observations in the blood-perfused airway wall is of fundamental importance here. Dead, apoptotic granulocytes cannot migrate. It is not likely, therefore, that the occurrence apoptotic leukocytes in the airway lumen can tell anything about apoptosis in the airway wall. What is then the role of apoptosis of the lumen cells in airways diseases? A widely quoted, uncontrolled study from 1996 [39] reported that steroid treatment increased the percentage of apoptotic eosinophils in the airway lumen. It was claimed that this action was important for the resolution of airway inflammation in asthma. In contrast, a subsequent placebo-controlled trial involving a high dose inhaled steroid found no increase in apoptotic eosinophils in sputum samples despite a reduction in sputum eosinophils [40]. In further contrast to predictions from in vitro findings, the number of sputum macrophages that had ingested eosinophils was reduced in the steroid-treated asthmatic individuals compared to placebo [40]. Inconclusive clinical observations of leukocyte apoptosis in the airway lumen have been reported not only in asthma [13,39-41] but also in COPD [13,41,42], cystic fibrosis [2,43], and bronchiectasis [44]. Matute-Bello and Martin [45], who originally discovered an anti-apoptotic action of BAL fluid in adult respiratory distress syndrome, have now argued that neutrophil apoptosis may have little to do with outcome. Findings in airway lumen in acute lung injury in newborn infants [46] may similarly disallow firm conclusions on roles of neutrophil apoptosis. Also, the hypothesis that the airway lumen milieu in COPD would promote neutrophil survival was not supported in studies where neutrophils were exposed to airway lumen fluids [42]. In summary, roles in disease for apoptosis and subsequent phagocytosis of apoptotic leukocytes in the airway lumen remain to be defined.

### Steroid-induced apoptosis of eosinophils, lymphocytes, and dendritic cells in the airway wall in vivo?

The relatively rapid steady-state turnover of airway mucosal dendritic cells (Holt et al 94) is considered to reflect the need for continuous immune surveillance and emigration of these cells to regional lymph nodes. There are increased numbers of bronchial mucosal dendritic cells in asthma and they are downregulated by prolonged steroid treatment (Möller et al96). Mechanisms involved in this drug-induced elimination of dendritic cells remain unknown although in rats receiving a large systemic steroid dose apoptosis is responsible in part for the rapid loss of tracheal mucosal dendritic cells (Brokaw 1998). In vitro studies on steroid-induced apoptosis of dendritic cells are scarce whereas steroid-induced apoptosis of eosinophils and lymphocytes [1,29,30] has received much attention. However, the reputed eosinophil apoptosis-inducing effect of glucocorticoids has not been borne out in in-vivo studies of airway tissues [8,11,13,26] nor could steroid-induced T cell apoptosis be consistently demonstrated in biopsies obtained from asthmatic [47,48] and COPD patients [49]. Indeed, compelling evidence now appears to be lacking to show that infiltrated eosinophils and lymphocytes are eliminated from the airway wall through apoptosis followed by phagocytosis. Animal and human airway wall eosinophils seem to increase and decline without occurrence of detectible apoptotic eosinophils, whether phagocytosed or not [7,8,13,27].

### Steroid-induced inhibition of neutrophil apoptosis in the airway wall in vivo?

Steroid-induced attenuation of neutrophil apoptosis was demonstrated in vitro in 1995 [50]. This effect on cultured cells has since been considered to explain observations in vivo of airway wall neutrophilia induced by steroid treatment in both COPD [51-53] and asthma [54-56]. However, as with steroid-induced increase in apoptosis of eosinophils and lymphocytes, the steroid-induced attenuation of neutrophil apoptosis has not been compellingly demonstrated in the diseased airway wall. Gizycki et al [51] examined the ultrastructure of neutrophils in COPD biopsy tissues. Since morphologic cell features accurately define apoptosis this technique should be ideal for assessing this fate of the neutrophils. However, no effect of steroid treatment on neutrophil apoptosis was observed. Gizycki et al concluded "the functional significance of the potential for steroids to reduce the clearance of neutrophils by their effect on apoptosis is unclear in vivo".

Little attention has been given to the alternative possibility that the steroid effect could reflect upregulation of neutrophil-retaining chemokines in the airway wall. Steroid treatment in mild asthma actually increases mucosal expression of major neutrophil attractants such as IL-8 and INF-gamma-inducible protein 10 [54]. In steroid-treated severe exacerbations of asthma, CXCL5-dependent mechanisms [56] may further contribute to recruiting and retaining the neutrophils in the airway wall. These actions alone suggest that steroids may reduce transepithelial migration of neutrophils. This possibility is now amply supported by clinical observations on cells in airway wall and lumen, respectively. Contrasting the steroid-induced airway wall neutrophilia [51-56] several human in vivo studies, involving steroid-treated COPD patients and other steroid-treated neutrophilic airway conditions [53,57-60], have now shown reduced airway lumen neutrophils. These reciprocal effects, convincingly confirmed in one and the same study [53], strongly suggest that steroid treatment reduces transepithelial migration of airway wall neutrophils. The significance of this steroid action is not known. However, it should be difficult to accept the conclusion that an anti-inflammatory effect of steroid treatment has been achieved merely based on reduced numbers of airway lumen neutrophils (51,52).

### Transepithelial migration of leukocytes without harming the epithelial lining

The passage of granulocytes such as eosinophils and neutrophils across the epithelium in asthma and COPD is thought to be part of pathogenic disease processes with a capacity to cause severe epithelial injury [15,61-63]. This view can be debated. For example, commonly obtained evidence of barrier dysfunction is based on reduced electrical resistance. However, the bioelectrical properties of the epithelial lining may not be equated with physiologically important barrier functions [64]. It is even possible that apical epithelial junction proteins including occludin can be reduced without undue effects on epithelial barrier function. Also, numerous eosinophils and neutrophils have been demonstrated to migrate into the airway lumen in animals and man in vivo without injuring the epithelium [16,65]. Thus, about 35 000 eosinophils per minute and per cm2 mucosal surface area transmigrated across a normal, human airway-like, guinea-pig tracheal epithelial lining in vivo leaving ultrastructurally intact epithelial apical cell to cell contacts [16]. Similarly, bronchial instillation of LTB4 in human subjects and LPS in sheep produced transmigration of neutrophils into the airway lumen without evidence of epithelial injury [65]. Importantly, the transepithelial exit of cells as well as extravasated plasma proteins occur without increasing epithelial permeability in the reverse direction. The swift entry of cells and macromolecules into the airway lumen, without increasing the absorption rate of luminal macromolecules, tells about the plasticity or valve-like function of para-epithelial junctions [14,65]. This epithelial feature also explains why exudative allergic and inflammatory airways diseases do not exhibit increased absorption permeability; until proven otherwise asthma and allergic rhinitis may rather be characterised by reduced absorption of inhaled molecules in vivo [14,66].

Granulocytes may seem guilty by their association with sites of epithelial injury. Yet, the relation could be the reverse in that epithelial cell injury can provide potent stimuli for recruiting activated neutrophils and eosinophils to the repair site and to the airway lumen [67]. Further work thus seems needed to determine under what circumstances a mere passage of leukocytes can harm the airway epithelial lining in health and disease. Studies are also warranted to better elucidate the non-injurious nature of transepithelial egression of leukocytes especially at resolution of airway inflammation.

### Transepithelial egression of inflammatory cells at clinical improvement of airways disease

#### Eosinophils

In animal models of allergic airway inflammation [11,13,26] airway lumen eosinophilia has occurred during resolution when eosinophils have disappeared from the airway wall. We have found three clinical experimental studies where both airway wall and lumen eosinophils have been determined during the resolution phase. These studies involved allergen-challenged subjects with mild allergic asthma, It seems highly significant that all three studies consistently (and "unexpectedly") demonstrate that loss of infiltrated bronchial mucosal tissue eosinophils is associated with increased numbers of eosinophils in the bronchial lumen [68-70]. In accord, during the resolution phase a significant negative correlation between airway wall and airway lumen eosinophils was observed [68].

#### Mast cells

In 1992 Juliusson and co-workers [71] made the interesting observation that the number of mast cells in allergen-challenged individuals with seasonal allergic rhinitis increased progressively in the nasal epithelium during ten hours following provocation. Importantly, with a delay of about two hours also the nasal airway lumen mast cells exhibited a progressive increase in numbers. As with many other aspects of nasal mucosal inflammatory responses [72] this transepithelial exit of mast cells could well reflect what would occur also in the allergic bronchi. Studies involving allergic asthmatics support this possibility. Crimi et al [73] reported a significant correlation (r = 0.8;p < 0.001) between the number of superficial mucosal mast cells in bronchial biopsies and the severity of a resolving allergen challenge-induced late phase asthmatic reaction. Furthermore, Gauvreau et al [74] then demonstrated that mast cell numbers in the bronchial lumen correlated with the magnitude of an allergen challenge-induced late phase reaction that had occurred many hours previously. These observations in human nasal and bronchial airways suggest that epithelial transmigration of mast cells is a facet of resolution of the allergic late phase airway inflammation.

#### Lymphocytes and dendritic cells

In agreement with the possibility that lymphocytes are eliminated by transepithelial migration [3] Lommatzsch et al [75] have demonstrated peak numbers of lymphocytes in the airway lumen during the resolution phase several hours post allergen challenge in asthmatics. Also dendritic cells may in part be eliminated by exit into the airway lumen. When sampling inhaled allergens dendritic cells must extend processes through the epithelial tight junction barrier while maintaining the tight seal [76]. However, following allergen exposure there is also a marked increase in fully transmigrated dendritic cells in the airway lumen in animals and man [77-79]. The exit into the lumen is not immediate but is evident several hours post challenge in patients with allergic asthma [79]. It is as yet unclear what role these cells may play in the airway lumen. It has been speculated that some lumen dendritic cells may sample allergen and migrate back into the mucosa and to regional lymph nodes and that some may maintain local secondary immune responses for prolonged times after allergen exposure. Although the possibility may not have been discussed previously, even when relatively marked increases in lumen dendritic cells have occurred, it cannot be excluded that exit of mature and immature dendritic cells into the airway lumen in asthma and COPD [79,80] represents a mode of elimination of these cells from the airways.

#### Neutrophils

Lommatzsch et al [75] have demonstrated that neutrophils exhibit peak numbers in the airway lumen during the resolution phase several hours post allergen challenge in asthmatics with mild disease. Otherwise it is severe asthma that is characterised by airway neutrophilia [81,82]. In a study involving patients with acute severe asthma requiring intubation, tracheal aspirates were obtained continuously until extubation [83]. "Unexpectedly", the clinical improvement in these patients was associated with a marked increase in the numbers of airway lumen neutrophils over several days until exubation. We have not found any reports that contradict this important finding. These data, obtained in resolving severe asthma, may rather be compared to the increase in airway lumen neutrophils and lymphocytes that occurs over several months in COPD along with clinical improvement after smoking cessation [84,85].

Since the transepithelial passage of leukocytes has been considered a pathogenic process these observations have remained unexplained. We submit that the above clinical data on occurrence in the airway lumen of a range of immune cells in mild and severe asthma and in COPD reflect the role of transepithelial exit as a mode of ridding diseased bronchial tissues of inflammatory cells.

### Altered transepithelial migration in chronic airways disease

An increased airway wall chemo-attraction would recruit granulocytes from the microcirculation but also retain these cells in the wall. In severe exacerbations of COPD up to a 100-fold upregulation of neutrophil chemoattractants in bronchial mucosal tissues may thus explain the airway wall neutrophilia in these patients [86]. It further appears that neutrophil chemo-attraction in the airway lumen in COPD is abnormally low [87] and that neutrophils in severe COPD exhibit reduced chemotaxis compared to neutrophils in mild COPD [87]. Thus, increased attraction and retention of neutrophils in the airway wall, together with reduced neutrophil migration ability, could act in concert to modulate trans-epithelial egression of these cells in severe COPD. At exacerbations of COPD, a patchy occurrence of infected or injured bronchial epithelial cells [67,88] could bring large numbers of neutrophils to the airway lumen. This is an important innate immunity response. Patients with COPD, who presented with exacerbations due to either bacterial or viral infection, also exhibited airway lumen neutrophilia [89].

In stable COPD it has been noted that neutrophilia in the airway lumen can be associated with lack of neutrophilia in the airway wall [82]. Similarly, a particular subgroup of COPD patients, who have bronchitic symptoms of chronic cough and expectoration, exhibited lower airway wall eosinophil counts and higher airway lumen eosinophils than subjects with COPD without chronic bronchitis [90]. Hence, chronic conditions with decreased leukocytes in the wall and increased leukocytes in the airway lumen may be characterised by a degree of accelerated transepithelial migration. It is possible that such patients will respond particularly well on treatments that stop recruitment of circulating inflammatory cells to the airway wall. Andersson et al [91] recently made the observation that the most severe stage of COPD was associated with reduced numbers of mast cells in the airway wall compared to less severe COPD. This difference could not be explained by increased apoptosis of airway wall mast cells but was associated with increased numbers of mast cells in the airway lumen [91]. Hence, similar to the resolution phase after allergen challenge in rhinitis [71] and asthma [73,74] airway wall mast cells seem to be eliminated by transmigration into the airway lumen in severe COPD.

### Preventing transepithelial migration of leukocytes aggravates inflammation

An acknowledged research paradigm advises that inducement of eosinophil apoptosis will be of benefit in allergic airway diseases. A seminal supporting study demonstrated FAS ligand-induced eosinophil apoptosis and reduced airway lumen eosinophilia in mice with allergic inflammation [92]. We asked whether these effects in the airway lumen also involved anti-inflammatory actions in the airway wall? They did not. Although FAS treatment did produce apoptotic eosinophils also in the airway wall, this effect was associated with much increased cellular inflammation in this important location [93]. Phagocytosis of the apoptotic cells was clearly insufficient. Hence, many granulocytes underwent necrosis in the airway wall [93]. This contributed to the aggravated inflammation. In addition, reduced trans-epithelial egression of granulocytes contributed to the increased airway wall inflammation. Reduced transmigration could also explain the reduced airway lumen eosinophilia that had been repeatedly demonstrated in such FAS-treated animals. Strengthening the resolving role of transepithelial cell migration more studies have demonstrated that inhibition of transepithelial migration of granulocytes causes severe airway symptoms in allergic mice. This effect has been seen by inhibition of ICAM-2 [94] and by knock-out of matrix metalloproteinases (MMP) 2 and 9 [95,96]. In rats with virus-induced inflammation, repeated low level allergen challenges produced persistent eosinophilia in the airway wall but not in the airway lumen. The exclusive airway wall eosinophilia was associated with loss of lung elastic recoil [97]. In contrast, the allergen-exposed control rats had fewer eosinophils in the airway wall but more eosinophils in the airway lumen. They also had no loss of elastic recoil [97]. Hence, viral infections may have impeded the transepithelial exit of inflammatory cells and thus increased the effects of allergen exposure on lung mechanics. This possibility is of interest in view of the role of viral infection in exacerbations of asthma and COPD [98-100]. Human studies are warranted to explore the role of impeded transepithelial egression of leukocytes in viral induced aggravation of inflammatory airways diseases. Inhibition of transmigration should also receive attention as a mechanism and strategy by which viruses can escape host immune surveillance and defence.

Recent attempts to produce beneficial effects in COPD and asthma by reducing the traffic of granulocytes include the use of CCR inhibitors [101]. However, the possibility that such drugs may reduce egression from the diseased airway wall needs to be considered. Giving an antibody to block interleukin-5, a regulator of eosinophilopoiesis, eosinophil migration, and eosinophil survival, eliminated both blood and airway lumen eosinophilia [102] but had little effect on airway wall eosinophils [103], and no pro-apoptotic effect has been demonstrated. It is possible that the persistent airway wall eosinophilia in this situation reflected an inhibitory effect on trans-epithelial cell migration by the antibody blocking interleukin-5. Stopping both recruitment and trans-epithelial exit will result in elimination of tissue eosinophils only after a considerable delay. The extent of the delay depends on the (little known) half-life of the leukocyte in airway tissues. Observations in bronchial biopsies obtained from anti-IL-5 treated patients [103] suggest a long half-life of the airway tissue dwelling eosinophils. This inference would agree with the recent finding that clinical efficacy of anti-IL-5 treatment can be obtained in severe asthma after treatment over relatively long periods of time [104,105].

### Pathways involved in transepithelial egression

The human airway mucosa harbours a profuse microvascular network of capillaries and venules that receive systemic blood. Characterising the nasal mucosa and stretching all the way from the trachea to the smallest bronchioli this microcirculation occupies the area just beneath the epithelial lining. Through transendothelial migration in post-capillary venules leukocytes can thus be effectively delivered to the human airway mucosa anywhere along the nasal passages and the tracheobronchial tree. Reviews in the field of leukocyte trafficking in alveoli and airway passages in man and mice [106] often stress the fact that the low pressure pulmonary circulation differs from systemic microvascular beds by a specific sequestering of leukocytes notably the neutrophil. Location (capillary vs venular) and mechanisms (integrin independent vs dependent) involved in the extravasation of leukocytes also differ [106]. However, it seems less appreciated that the intrapulmonary airways in mice actually lack a systemic bronchial circulation [107]. Instead, these murine bronchi are fed by the pulmonary circulation. This adds to the shortcomings of the mouse models in their endeavour to mimic human asthma and COPD [108]. We have included mouse in vivo data in this review because here the focus is on the fate of the leukocytes after they have left the blood stream. Future experiments are warranted to provide data on the origin of the airway lumen leukocytes. Which vascular system has delivered them and from which part of the tracheo-bronchial-alveolar tree have they migrated?

**Figure 1:**
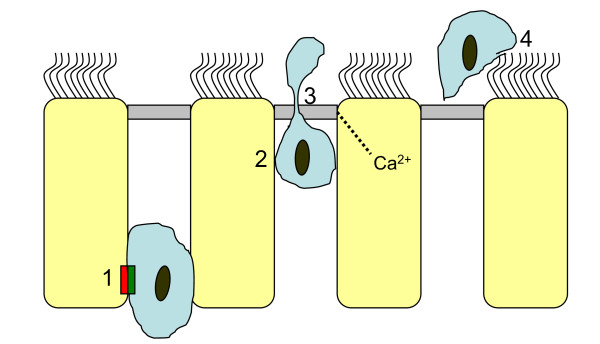
**"Schematic representation of trans-epithelial loss of leukocytes into airway lumen"**. Modified from references [61,16,112]. This scheme identifies some of the steps where future research is warranted to delineate mechanisms involved in the trans-epithelial elimination of inflammatory cells from the airway wall. 1 After cell-to-cell contact at the epithelial base paraepithelial crawling of the leukocyte may begin by integrin binding to desmosomal junction adhesion molecules. 2 Several binding interactions and cellular signalling events including cytosolic Ca++ fluxes may be involved as the leukocyte continues to migrate between juxtapositioned epithelial cells. 3 Binding interactions involving junction adhesion-like proteins and receptors such as the coxsackie and adenovirus receptor may be involved in protein-tight passage of the leukocyte through the tight apical junction complex. 4 After its elimination from the airway wall the leukocyte mixes with epithelial lining fluids and is finally eliminated by mucociliary clearance.

After extravasation, leukocytes move through interstitial tissue components [109] and through the epithelial basement membrane [110]. They attach to the basal epithelium and move through relatively long stretches of epithelial cell junction complexes. Recent reviews provide updates on mechanisms and potential pathogenic roles of transepithelial migration of neutrophils [15,61,111-113]. The focus is generally on in vitro observations and the data have not been generated to shed light on elimination of airway tissue leukocytes at resolving inflammation. There is information on in vivo alveolar epithelial passage of neutrophils in the excellent review by Burns et al [111] but little is known about corresponding bronchial epithelial mechanisms. Analogous to trans-endothelial migration of leukocytes the trans-epithelial migration can be grossly viewed as a three-step event: adhesion to the barrier cells, paracellular passage, and postmigration fate [114]. In many details, however, mechanisms concerning the passage of leukocytes across the venular endothelial lining may not be similar to mechanisms regulating the transepithelial egression of leukocytes. A major difference is that the two crossings are in opposite directions: the venular para-endothelial exit is apical to basal and the epithelial passage is basal to apical. Although little is known in detail about airway epithelial transmigration of leukocytes at resolution of inflammation, some general aspects may be illustrated (Figure 1).

#### Neutrophils

With intestinal epithelial cells in vitro as a model, Parkos and colleagues [61,114] have described several molecular interactions between neutrophils and epithelial cells during egression from the epithelial base to the lumen surface of the epithelium. These authors also focused on pathogenic effects of the transmigration. Through CD11b/CD18 interactions with epithelial counter-receptors neutrophils adhere to the basolateral aspect of epithelial cells. The further para-epithelial passage is facilitated by a series of events opening and closing the apical junction complex. A number of adhesive interactions of neutrophils with epithelial intercellular junction proteins occur during the paracellular migration. Then ICAM-1 expressed by the apical epithelial membrane may serve as a ligand for CD11b/CD18 to keep the transmigrated neutrophil attached to the luminal surface of the epithelial lining. This tethering action could be desirable in mucosal defence but is probably not suited for the clearance of cells away from inflamed airway tissues. Importantly, intestinal epithelial cells seem to differ from airway epithelium by exhibiting barrier damage in association with neutrophil transmigration [112].

#### Eosinophils

By creating transepithelial chemokine gradients, MMP 2 and 9 may produce transepithelial loss of lung parenchymal eosinophils and other leukocytes in allergic mice [95,96]. Increased lumen levels of CCL11 have been associated with acute loss of mucosal eosinophils into the tracheal lumen in allergen-challenged guinea-pigs [115]. In vitro observations further suggest that eotaxin-3, produced by IL-4 stimulated airway epithelial cells, and eosinophil expression of CCR3 mediate transepithelial migration [19]. CCL5 may also contribute. TNF-alpha may promote transepithelial migration in vitro of both eosinophils and neutrophils whereas IL-4 increased eosinophil but reduced neutrophil transepithelial migration [19].

#### Lymphocytes

Porter and colleagues [3,17] demonstrated non-injurious migration of lymphocytes across human cultured bronchial epithelium. They suggested that polarized epithelial localisation of chemokine ligands, including CCXCL10 [116] and CXCL11, to the epithelial apex determined elimination of CCR7+ T-lymphocytes from the airway wall [17]. Whereas these ligands may operate in COPD [17,116], previous workers have suggested that polarized epithelial localisation of CCL5 may regulate transepithelial migration of lymphocytes in asthmatic bronchi [117]. "Chemorepellents" [118,119] might aid the trans-epithelial exit of lymphocytes and other leukocytes. In accord with this possibility, Caulfield et al [120] have suggested that steroid-induced up-regulation of CXCR4 receptors may move leukocytes away from inflamed airways in asthma.

### Clearance of cells from the airway lumen is essential

The potential role of trans-epithelial egression in resolution of inflammation underlines the importance of cell clearance from the airway lumen. Clearing airway wall leukocytes across the nasal [72] and bronchial epithelium may be followed by swift and uneventful final elimination from the lumen. Due to the lack of a mucociliary escalator and lack of effect of coughing, clearance of leukocytes across the alveolar epithelium may be more problematic. Yet, in studies of lung inflammation in mice it appears that egression of parenchymal leukocytes into the alveolar air space is significantly beneficial; when this trans-epithelial egression was prevented severe asphyxia resulted [95,96]. We need to know to what extent apoptosis-related mechanisms can effect clearance of cellular exudates from the bronchial as well as the alveolar lumen. Interventions that can improve mucociliary clearance [121-125] need increased attention. It is particularly important that human peripheral airways can be freed from leukocyte- and plasma protein-rich exudates that otherwise will contribute to small airway closure [14,126,127].

### Use of sputum cell counts to adjust treatment

Analysis of induced sputum has advantages over determination of exhaled NO that recently was deemed to be of little value as a guide to treatment interventions in asthma [128]. Studies involving sequential sampling of sputum in stable disease suggest that leukocyte counts in induced sputum samples may exhibit acceptable repeatability [129,130]. These data support the use of induced sputum in monitoring disease severity and evaluating anti-inflammatory treatments in stable asthma and COPD. Of significant interest is the possibility that sputum indices can predict disease exacerbations. This is an area where sputum analysis has fared better than a clinical strategy involving symptoms and spirometry [131-135]. Since the first successful study of inhaled steroids half a century ago sputum eosinophilia has shown its value in predicting which patients will benefit from treatment with these drugs. In a matter of days to weeks after instituting steroid treatment both airway wall and lumen eosinophils will be much reduced. Adjusting the steroid dose to keep sputum eosinophil counts low successfully reduces the exacerbation rate in asthma [131-133]. However, as stated by Jayaram et al [133] "the observation that treatment to control sputum eosinophilia reduced eosinophilic exacerbations may not be a surprise, since treatment was designed to prevent these" (in this case by keeping sputum eosinophils < 2%). It might also be expected that the exacerbations that follow from tapering steroid doses can be predicted by sputum eosinophils[134,135]. Interestingly, loss of control of asthma following rapid withdrawal of steroids was associated with increased sputum neutrophils [136]. Reduced sputum neutrophilia was also helpful as an index of therapeutic effects of clarithromycin in refractory asthma [137]. It is of note that sputum data, even better than bronchial biopsy data, have identified individuals with regard to risks for exacerbation [138]. This observation may reflect the fact that sputum samples represent cumulative events over a large surface area involving also more peripheral airways than those available to biopsies. At growing inflammation, the airway wall is increasingly infiltrated with cells. A portion of these cells will migrate into the lumen. In this situation the epithelial transmigration does not reflect a resolving airway inflammation. However, a 'spill-over' of cells would be recorded in sputum samples as a sign of an arriving exacerbation. Future studies specifically addressing the relationship between airway lumen and airway wall eosinophils in developing exacerbations are warranted to further elucidate this possibility.

Leukocytes in the bronchial lumen in asthma and COPD may differ between large and small bronchiolar-alveolar airways. In accord there are differences as regards the relative proportions of different leukocytes occurring in sputum specimens and broncho-alveolar lavage (BAL) fluids, respectively [139,140]. This is a concern since much of the pathology of asthma and COPD resides in the small airways. Tillie-Leblond and colleagues [141] further noted that only half of ten studies on the subject could demonstrate a relationship between eosinophils in induced sputum samples and symptoms of asthma. Caution is also advised in interpretation of sputum data since airway tissue and lumen may differ as to which granulocyte, eosinophil or neutrophil [81,82], and which T lymphocyte, especially Tc1 or Tc2 [116,142-144], is predominant. Irrespective of such differences, it is commonly assumed that numbers of leukocytes in sputum samples reflect intensity of cell-mediated inflammatory processes in diseased bronchial tissues. The present hypothesis infers that the timing of obtaining samples in relation to developing and resolving disease conditions is crucial. Thus, during development of inflammation the cell content of sputum samples may underestimate bronchial tissue cellularity. Reversely, during an active resolution phase when cells are being eliminated from the airway wall the sputum samples could grossly overstate the numbers of airway wall cells. Awareness of this confounding possibility may improve interpretation of sputum data.

We have introduced a dual induction method [66] whereby inhalation of histamine first induces a prompt bronchial plasma exudation response. About an hour later a second induction, this time of sputum, is employed. The induced sputum then retrieves the exuded plasma together with other mucosal interstitial proteins that the travelling plasma may have picked up. This technique can improve the protein yield of induced sputum and be employed to examine the pharmacology of plasma exudation and the occurrence of exudative hyperresponsiveness. Although the laying down of exuded plasma proteins (including fibronectin and fibrin) may pave the way for cell traffic cells cannot be expected to migrate into the airway lumen along with the bulk plasma. Perhaps other inhalational challenges than histamine can be developed that safely will bring cells into the airway lumen to improve the cellular yield of a subsequent sputum induction. As a bonus this work could lead to discovery of interventions that will speed up resolution of airway wall inflammation.

## Conclusion

We have argued here that the occurrence of eosinophils, neutrophils, lymphocytes, and mast cells in the bronchial lumen can reflect their successful and non-injurious elimination away from cell-mediated disease areas in the airway wall. Evidence obtained in animal models together with a large variety of clinical observations, previously considered unexpected, support the importance of egression as a mode of eliminating pro-inflammatory leukocytes from diseased airway tissues. These clinical reports have been publicised during the last two decades. Simultaneously, the central role of leukocyte apoptosis in resolution of airway diseases that we and others have been seeking has not been confirmed. The possibility of resolution through transepithelial exit of cells needs consideration in studies of airway diseases and when assessing the effects of drug interventions. Otherwise, data on airway lumen leukocytes alone can lead to paradoxical conclusions. Inhibiting pro-inflammatory, inciting processes in the airways is important and so is rapid and complete healing of epithelial injury [67]. However, it may not suffice to reduce recruitment of inflammatory leukocytes to the airway wall. We suggest that additional effects of promoting transepithelial migration, together with a secured clearance of cells from the airway lumen, are important for accomplishing resolution of cell-mediated airways diseases.

## Authors' contributions

CP prepared the first draft. LU contributed several versions, made the figure, and both approved the final manuscript.

## Competing interests

The authors declare that they have no competing interests.
